# DNA Binding Test, X-Ray Crystal Structure, Spectral Studies, TG-DTA, and Electrochemistry of [CoX_**2**_(dmdphphen)] (Dmdphphen Is 2,9-Dimethyl-4,7-diphenyl-1,10-phenanthroline, X = Cl, and NCS) Complexes

**DOI:** 10.1155/2014/914241

**Published:** 2014-12-29

**Authors:** Mousa Al-Noaimi, Mohammed Suleiman, Hany W. Darwish, Ahmed H. Bakheit, Muneer Abdoh, Iyad Saadeddin, Naveen Shivalingegowda, Neartur Krishnappagowda Lokanath, Odey Bsharat, Assem Barakat, Ismail Warad

**Affiliations:** ^1^Department of Chemistry, Hashemite University, Zarqa 13115, Jordan; ^2^Department of Chemistry, Science College, An-Najah National University, P.O. Box 7, Nablus, Palestine; ^3^Department of Pharmaceutical Chemistry, College of Pharmacy, King Saud University, P.O. Box 2457, Riyadh 11451, Saudi Arabia; ^4^Department of Analytical Chemistry, Faculty of Pharmacy, Cairo University, Kasr El-Aini Street, Cairo 11562, Egypt; ^5^Department of Physics, Science College, An-Najah National University, P.O. Box 7, Nablus, Palestine; ^6^Institution of Excellence, Vijnana Bhavan, University of Mysore, Manasagangotri, Mysore 570 006, India; ^7^Department of Studies in Physics, University of Mysore, Manasagangotri, Mysore 570 006, India; ^8^Department of Chemistry, College of Science, King Saud University, P.O. Box 2455, Riyadh 11451, Saudi Arabia; ^9^Department of Chemistry, Faculty of Science, Alexandria University, P.O. Box 426, Ibrahimia, Alexandria 21321, Egypt

## Abstract

Two new neutral mixed-ligand cobalt(II) complexes, [CoCl_2_(dmdphphen)] **1** and [Co(NCS)_2_(dmdphphen)]  **2**, where dmdphphen is 2,9-dimethyl-4,7-diphenyl-1,10-phenanthroline, were synthesized and characterized by an elemental analysis, UV-Vis, IR, TG/DTA, cyclic voltammetry CV, and single X-ray diffraction. Complex **2** crystallized as monoclinic with a space group P2_1_/c. Co(II) ions are located in a distorted tetrahedral environment. TG/DTA result shows that these complexes are very stable and decomposed through one-step reaction. The two complexes exhibit a quasireversible one-electron response at −550 and 580 mV versus Cp_2_Fe/Cp_2_Fe^+^, which has been assigned to Co(I)/Co(II) and Co(II)/Co(III) couples. Absorption spectral studies reveal that such complexes exhibit hypochromicity during their interaction with CT-DNA.

## 1. Introduction

1,10-Phenanthroline ligands and their derivatives are very attractive in metal complexes [1–3]. In addition, their metal complexes are frequently used as catalyst for the enantioselective hydrolysis of *N*-protected amino acid esters, allylic substitutions, reduction of acetophenone [3–6], and oxidation of olefins [7]. Also, cobalt(II) complexes with a reversible Co(II)/Co(III) are a good oxygen carrier and can oxidize the double bond of the olefins [8–10].

The ability of the cobalt phenanthroline complexes to bind and to cleave DNA under physiological conditions is of current interest because of their potential applications in nucleic acids chemistry [11]. Also, these complexes are useful in footprinting studies [12–17]. The cleavage of DNA usually occurs through the heterocyclic bases, deoxyribose sugar moiety, or phosphodiester linkage [18–20]. For the mixed-ligand complexes to interact efficiently with DNA, the ligands need to be flat, have large surface area, and have a spatial geometry to interact with the base pairs in DNA [15–23]. By changing the ligands or the metal ions, it is possible to modify the interaction with nucleic acids [23–26].

Previously, a series of several mixed-ligand mononuclear [27, 28] and dinuclear [29, 30] metal complexes have a general formula MX_2_(dmphen) (2,9-dimethyl-1,10-phenanthroline, X = Cl, NCS) prepared in our lab. These complexes were found to be suitable precursors for spherical shape metal oxide nanoparticles [31]. Herein, two new neutral mixed-ligand cobalt(II) complexes, [CoCl_2_(dmdphphen)]** 1** and [Co(NCS)_2_(dmdphphen)]** 2,** where dmdphphen is (2,9-dimethyl-4,7-diphenyl-1,10-phenanthroline), were synthesized and characterized by different spectroscopic methods. Also, the DNA binding and the catalytic oxidation of styrene in the presence of H_2_O_2_ for the complexes were investigated.

## 2. Experimental Section

### 2.1. Materials and Instrumentation

2,9-Dimethyl-4,7-diphenyl-1,10-phenanthroline ligand, CoCl_2_·6H_2_O, and Co(NCS)_2_ were purchased from Acros Organics. Elemental analyses were carried out on an Elementar vario EL analyzer. The IR spectra for samples were recorded using PerkinElmer Spectrum 1000 FT-IR Spectrometer. The UV-Vis spectra were measured by using a TU-1901 double-beam UV-Vis spectrophotometer. TG/DTA spectra were measured by using a TGA-7 PerkinElmer thermogravimetric analyzer. The cyclic voltammograms for the complexes were measured in CH_3_CN and 0.1 M tetrabutylammonium hexafluorophosphate (TBAHF) using BAS 100 B/W electrochemical workstation (Bioanalytical Systems, West Lafayette, IN, USA) and controlled by a standard 80486 personal computer (BAS control program version 2.0). All electrochemical experiments were carried out at room temperature under argon with a three-electrode cell. Voltalab 80 potentiostat PGZ402 with Pt-disk electrode (Metrohm, *A* = 0.0064 cm^2^) was used as working electrode. Platinum wire (£ 1 mm) spiral with diameter 7 mm was used as a counter electrode. Haber-Luggin double reference electrode was used as a reference one. All potentials in this paper are reported to an external Cp_2_Fe^0/+^ standard [32].

### 2.2. General Procedure for the Preparation of the Desired Complexes

A mixture of CoX_2_ salt (2 mmol) in distilled ethanol (15 mL) and free ligand (2.1 mmol) in methanol (10 mL) is stirred for around 0.5 h at room temperature until the precipitation appeared which was filtered, washed with ethanol, and dried. Suitable crystals for X-ray diffraction analysis were growing up by slow diffusion of ethanol into a solution of the complex in CH_2_Cl_2_ after two days (yield 88%).

#### 2.2.1. Complex **1**

Yield: 0.76 g (90%). Anal. Calc. for C_26_H_20_Cl_2_CoN_2_: C, 63.69; H, 4.11; N, 5.71.* Found*. C, 63.43; H, 4.21; N, 5.48. UV-Vis (nm) bands in dichloromethane: 655, 572, 360, 240, 280 and 304. M.p 320°C. Conductivity in CH_3_CN: 10.28 (*µ*S/cm).

#### 2.2.2. Complex **2**

Yield: 0.94 g (88%). C_28_H_20_CoN_4_S_2_: Cal. C, 62.80; H, 3.76; N, 10.46; S, 11.97. Found. C, 62.92; H, 3.85; N, 10.33; S, 11.86. UV-Vis (nm) bands in dichloromethane: 645, 566, 358, 242, 282 and 305. M.p 290°C. Conductivity in CH_3_CN: 9.52 (*µ*S/cm).

### 2.3. Crystallography

A suitable single-crystal complex** 2** with dimensions of 0.23 × 0.22 × 0.21 mm was chosen for an X-ray diffraction measurement. X-ray intensity data were collected at 296 K on a Bruker CCD diffractometer equipped with Cu K_*α*_ radiation (*l* = 1.54178 Å). Data were collected with the *φ* and *ω* scan method. The final unit cell parameters were based on all reflections. Data reduction of all the collected reflections and absorption correction were carried out using the APEX 2 [33] package. The structure was solved by direct methods using SHELXS [34]. The structure was then refined by a full-matrix least-squares method with anisotropic temperature factors for nonhydrogen atoms using SHELXL [34]. All the nonhydrogen atoms were revealed in the first Fourier map itself. After several cycles of refinement, the final difference Fourier map showed peaks of no chemical significance and the residual saturated to 0.0671. Details of data collection and refinement are given in Table 1. The geometrical calculations were carried out using the program* PLATON* [35]. The molecular and packing diagrams were generated using the software* MERCURY* [36].

### 2.4. DNA Binding and Cleavage Experiments

Absorbance measurement was performed to clarify the binding affinity of cobalt(II) complexes by emissive titration at room temperature. The complexes were dissolved in mixed solvent of Tris-HCl buffer (5 mM Tris-HCl/50 mM NaCl buffer for pH = 7.2) for all the experiments and stored at 4°C for further use and used within 2 days. Tris-HCl buffer was subtracted through baseline correction. The absorption experiments were performed by keeping the concentration of cobalt(II) complexes constant (1.5 × 10^−4^ mol/L) and increasing the concentration of DNA gradually (1.0 × 10^−4^–1 × 10^−3^ mol/L).

## 3. Results and Discussion

### 3.1. Synthesis of the Desired Complexes

**Scheme 1:**
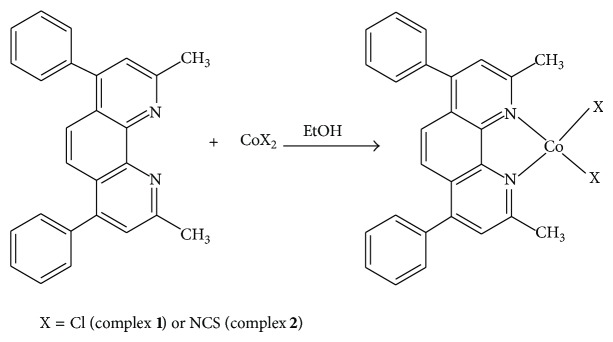
Synthesis of the Co(II) complexes** 1 **and** 2**.

The mononuclear CoCl_2_(dmdphphen) complex** 1** and Co(NCS)_2_(dmdphphen) complex** 2 **were isolated in a good yield without side product as seen in Scheme 1.

The structures of the desired complexes were confirmed by using elemental analysis, IR, UV-Vis, TG/DTA, and X-ray single-crystal measurement for complex** 2**. The analytical data of the complexes show the formation of [1 : 1 : 2] [M : dmdphphen : 2X] ratio in a good agreement with the suggested formula [CoX_2_(dmdphphen)] of the isolated complexes. The isolated solid complexes are insoluble in water, ethanol, *n*-hexane, and ethers but soluble in chlorinated solvents as CHCl_3_ and CH_2_Cl_2_. The solubility and molar conductance showed that the two complexes are nonelectrolytic in their nature.

### 3.2. X-Ray Crystal Structure

**Figure 1:**
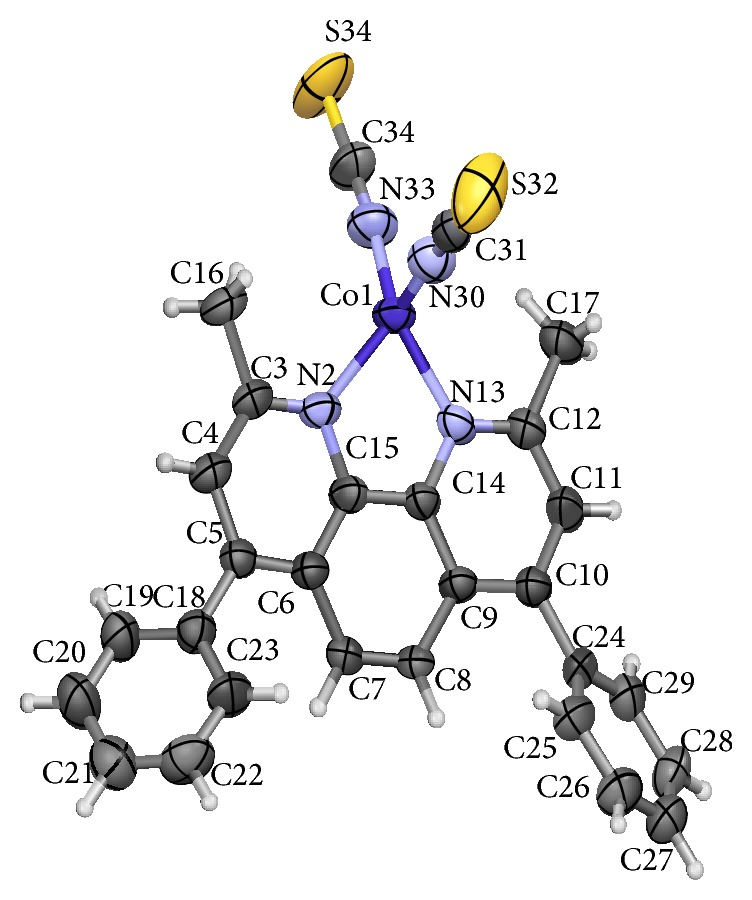
ORTEP of the complex** 2** with atom labelling. Thermal ellipsoids are drawn at the 50% probability level.

**Figure 2:**
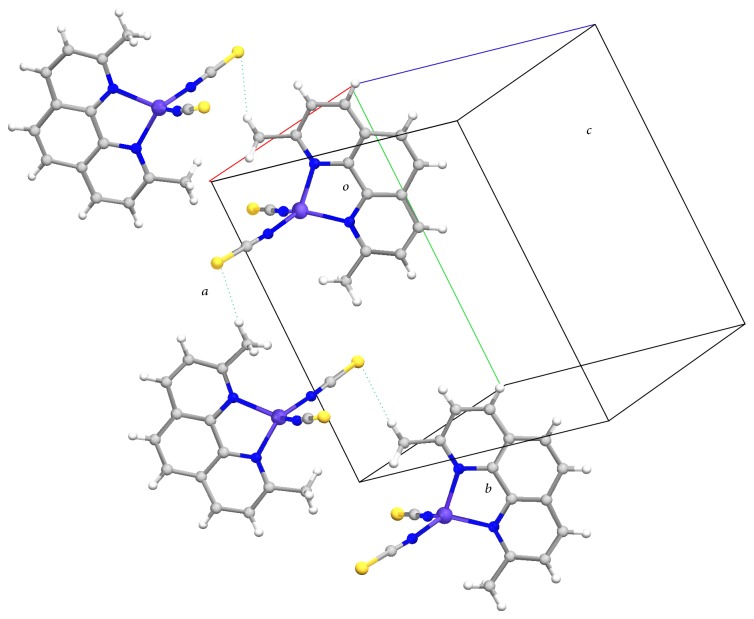
A crystal packing of complex** 2** viewed (perspective) along crystallographic *a* direction.

Crystal structure data and selected bonds length for complex** 2** are compiled in Tables 1 and 2, respectively. ORTEP drawing of the complex is shown in Figure 1. The central cobalt metal ion is coordinated to the two nitrogen atoms (N2 and N13) of the dmdphphen ligand and to two nitrogen atoms (N30 and N33) of the isothiocyanate ligand in a tetrahedral symmetry. The phenanthroline ring in the dmdphphen moiety is essentially planar with an rms deviation of 0.0752 Å. The phenyl rings (C8–C13 and C24–C29) are twisted out of the plane of the dmdphphen moiety as indicated by the dihedral angle values of 43.2(4)° and 47.2(4)°, respectively. All coordination distances and bond angles are similar to those found in similar compounds [37]. No classic hydrogen bonds were observed. In the crystal structure there is a *π*-*π* stacking interaction between adjacent dmdphphen and distances 3.7109(17) Å and 3.8070(17) Å, which may account for stabilizing the crystal structure (Figure 2). The packing of the molecules when viewed down along the *a* axis indicates that the molecules are interlinked by weak hydrogen bonds to form one dimensional chain.

### 3.3. IR Spectrum

**Figure 3:**
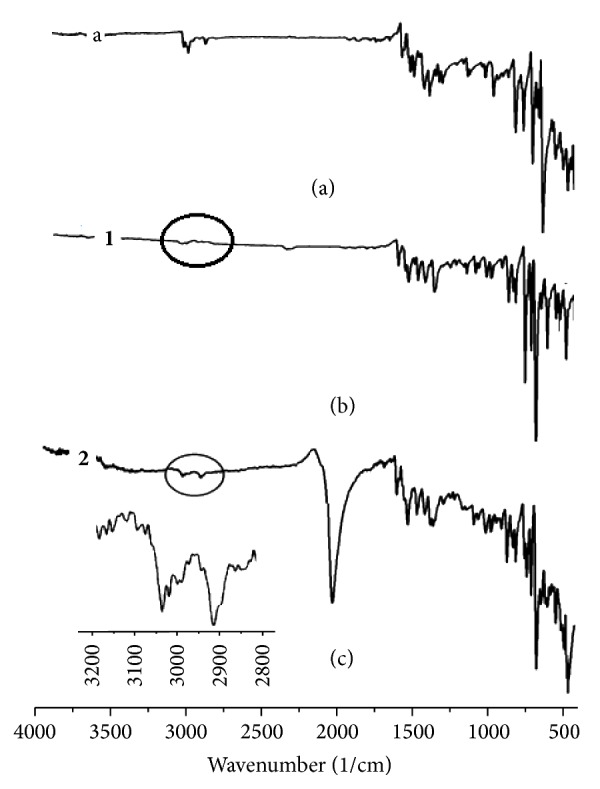
IR-KBr disk spectra of free ligand (a) and their desired complexes** 1 **and** 2**.

The IR spectrum of complex** 1** (Figure 3(b)) showed four characteristic absorptions peaks in the range of 3060, 2950, 550, and 350 cm^−1^ [7–10] which was assigned to H–Ph, H–CH_3_, Co–N, and Co–Cl stretching vibrations, respectively. New band at 2150 cm^−1^ which was assigned to NCS vibrations was observed in IR spectrum of complex** 2** (Figure 3(c)). The H–Ph, H of CH_3_ in dmdphphen bands appeared in their expected areas (Figure 3(a)).

### 3.4. Electronic Absorption Spectral Study

**Figure 4:**
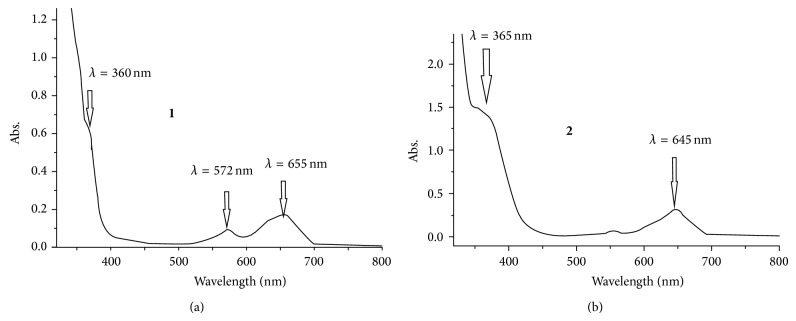
UV-Vis spectrum of the desired complexes (**1** and** 2**) in dichloromethane at RT.

The experimental absorption spectra (UV-Vis) of the [CoX_2_(dmphen)] complexes** 1**,** 2** in dichloromethane solution presented three dominant bands in the regions 200–800 nm (Figure 4). The bands in the UV region centered at around 240, 280, and 300 nm were assigned ligand-centered *π*-*π* ^*^ transitions (in both complexes). The bands at 360, 572, and 655 nm for complex** 1** (above) and at 365, 560, and 645 nm for complex** 1** (down) can be assigned to the d → d transition and MLCT, respectively [14–20].

### 3.5. Thermal Decomposition Analysis of Complexes **1**

**Figure 5:**
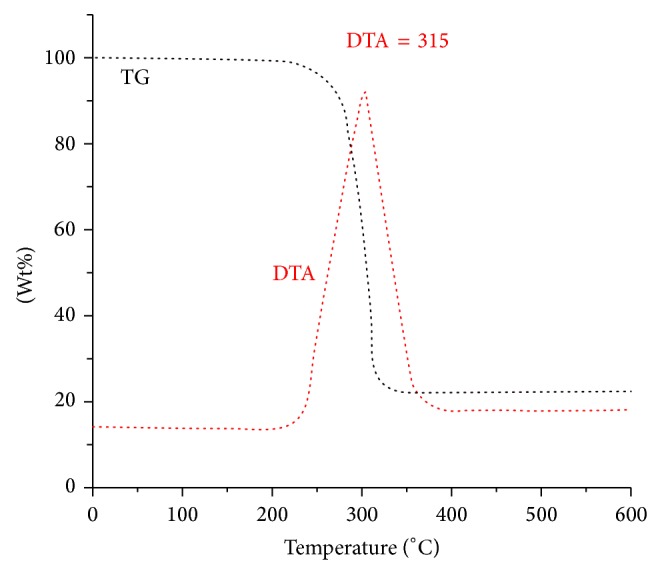
TG/DTA thermal curves of the desired complex** 1.**

The thermal analysis of complex** 1** (Figure 5) was investigated in the range of 0–600°C and heating rate of 10°C/min. Figure 5 shows that there is no uncoordinated or coordinated water in the range of 0–150°C and 150–180°C, respectively. Also, it shows that there are no decomposition intermediate steps of the coordinated chloride and dmdphphen ligands; both inorganic and organic ligands were destructured away from the Co metal with one-step broad decomposition in 200–330°C with weight loss ~81% and an exothermic DTA signal at ~315°C; the final residue was confirmed by IR to be CoO.

### 3.6. Electrochemistry

**Figure 6:**
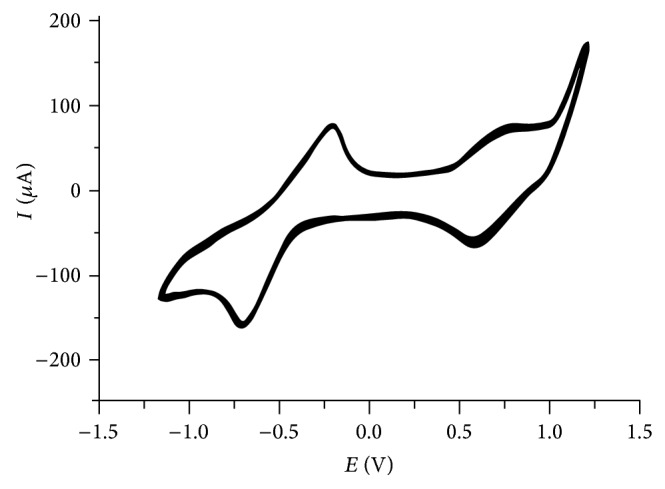
Voltammogram diagram of complex** 2** (*c* = 1 × 10^−3^ M, in acetonitrile solution, 0.1 M TBAHF, scan rate 100 mV/s at RT).

The electron-transfer behavior of the complexes in acetonitrile solution was examined by cyclic voltammetry. As a representative example, the cyclic voltammogram for complex** 2 **is shown in Figure 6. Complex** 2** exhibited two single electron reversible oxidative responses at −550 and 580 mV versus Cp_2_Fe/Cp_2_Fe^+^, which has been assigned to Co(I)/Co(II) and Co(II)/Co(III) couples, respectively. The dmdphphen ligand is electroinactive over the studied range of +1.5 to −1.5 V. Both complexes exhibit the similar behavior during the cyclic voltammetry experiments.

### 3.7. DNA-Complex **1** Binding Test

**Figure 7:**
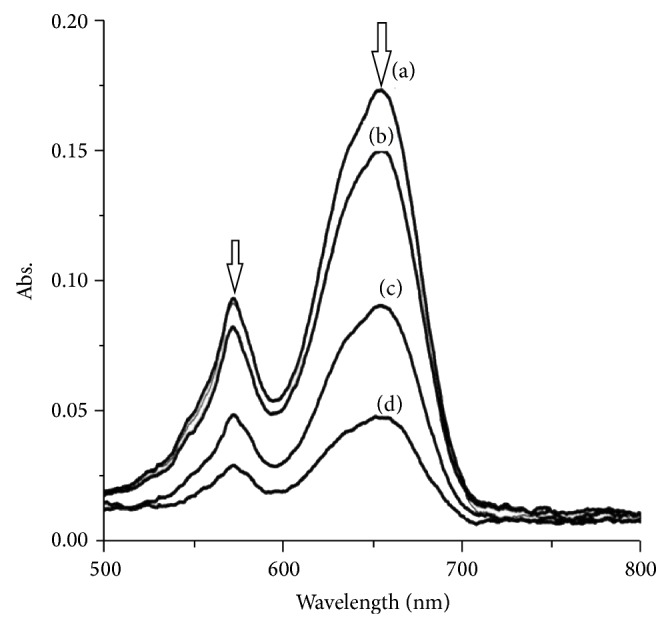
Visible spectra of 1.5 × 10^−4^ mol/L of complex** 1 **interacting with (a)—0, (b)—1.0 × 10^−4^, (c)—5 × 10^−4^, and (d)—1 × 10^−3^ mol/L CT-DNA at RT.

The affinity of Co(II) complexes for double-stranded CT-DNA was explored using UV-Vis titrations in deionised water. The results of representative titrations are shown in Figure 7. Complex** 1** showed good DNA binding affinity. Complex** 1** has three characteristic absorption peaks at 360 nm, 572 nm, and 655 nm, respectively. There is a decrease in an intensity for all peaks for complex** 1** by adding several concentrations of DNA. This suggests that the cobalt complex might be bind to DNA by an intercalative mode [38]. However, by comparing the small shift for complex** 1** with 7 nm red-shift values for Os(phen)_2_(dppz)^2+^ [39] and 9 nm for [Co(phen)_2_(pdtp)]^3+^ [40], this demonstrates that the intercalative strength of such complexes into DNA is not very strong.

## 4. Conclusions

Tetrahedral cobalt(II) complexes [CoCl_2_(dmdphphen)]** 1 **and [Co(NCS)_2_(dmdphphen)]** 2** were made available in good yield. Complex** 2 **was solved by XRD as monoclinic with a space group P2_1_/c. Co(II) ions are located in a distorted tetrahedral environment. TG/DTA result shows that these complexes are very stable and decomposed through one-step reaction; the complexes exhibit a quasireversible one-electron response at ~−550 mV assigned to Co(I)/Co(II) and ~580 mV assigned to Co(II)/Co(III) versus Cp_2_Fe/Cp_2_Fe^+^. Absorption spectral studies reveal that such complexes exhibit good DNA binding.

## Figures and Tables

**Table 1:** Crystal data and structure refinement for ligand and complex **2**.

	Complex **2**
Empirical formula	C_28_H_20_N_4_S_2_Co
Formula weight	535.55
Temperature	293(2) K
Wavelength	1.54178 Å
Crystal system	Monoclinic
Space group	P2_1_/c
Unit cell dimensions	*a* = 14.8373(12) Å *α* = 90° *b* = 21.0942(11) Å *β* = 100.191(4)° *c* = 8.2470(6) Å γ = 90°
Volume	2540.4(3) Å^3^
*Z*	4
Density (calculated)	1.400 Mg/m^3^
Absorption coefficient	7.017 mm^−1^
*F*(000)	1100
Crystal size	0.30 × 0.25 × 0.15 mm^3^
Theta range for data collection	3.03° to 63.94°
Index ranges	−15 ≤ *h* ≤ 17, −21 ≤ *k* ≤ 24, −9 ≤ l ≤ 6
Reflections collected	8247
Independent reflections	3934 [*R*(int) = 0.0671]
Refinement method	Full-matrix least-squares on *F* ^2^
Data/restraints/parameters	3934/0/318
Goodness-of-fit on *F* ^2^	1.047
Final *R* indices [*I* > 2sigma(*I*)]	*R*1 = 0.0671, *wR*2 = 0.1910
*R* indices (all data)	*R*1 = 0.1715, *wR*2 = 0.2661
Largest diff. peak and hole	0.641 and −0.870 e·Å^−3^

**Table 2:** Selected bond distances (Å) and bond angles (°) of the complex **2**.

Bond angles (°)		Bond distances (Å)	
N2–Co1–N13	82.11(18)	Co1–N13	2.035(4)
N2–Co1–N30	108.7(2)	Co1–N2	2.032(4)
N2–Co1–N33	122.3(2)	Co1–N30	1.923(7)
N13–Co1–N30	109.3(2)	Co1–N33	1.905(5)
N13–Co1–N33	122.8(2)	N2–C15	1.374(6)
N30–Co1–N33	109.0(2)	N13–C14	1.360(6)

## Additional Material

Crystallographic data for complex** 1 **has been deposited with the Cambridge Crystallographic Data Centre as supplementary publication number CCDC 1014019. Copies of this information may be obtained free of charge via https://www.ccdc.cam.ac.uk/conts/retrieving.html (or from the CCDC, 12 Union Road, Cambridge CB2 1EZ, UK; fax +44-1223-336033; e-mail deposit@ccdc.cam.ac.uk).
